# *In Vitro* Efficient Transfection by CM_18_-Tat_11_ Hybrid Peptide: A New Tool for Gene-Delivery Applications

**DOI:** 10.1371/journal.pone.0070108

**Published:** 2013-07-29

**Authors:** Fabrizio Salomone, Francesco Cardarelli, Giovanni Signore, Claudia Boccardi, Fabio Beltram

**Affiliations:** 1 NEST, Scuola Normale Superiore and Istituto Nanoscienze-CNR, Pisa, Italy; 2 Center for Nanotechnology Innovation @NEST, Istituto Italiano di Tecnologia, Pisa, Italy; Consejo Superior de Investigaciones Cientificas, Spain

## Abstract

Cell penetrating peptides (CPPs) are actively researched as non-viral molecular carriers for the controlled delivery of nucleic acids into cells, but widespread application is severely hampered by their trapping into endosomes. Here we show that the recently introduced endosomolytic CM_18_-Tat_11_ hybrid peptide (KWKLFKKIGAVLKVLTTG-YGRKKRRQRRR, residues 1-7 of Cecropin-A, 2-12 of Melittin, and 47-57 of HIV-1 Tat protein) can be exploited to obtain a self-assembled peptide-DNA vector which maintains the CM_18_-Tat_11_ ability to enter cells and destabilize vesicular membranes, concomitantly yielding high DNA transfection efficiency with no detectable cytotoxic effects. Different peptide-DNA stoichiometric ratios were tested to optimize vector size, charge, and stability characteristics. The transfection efficiency of selected candidates is quantitatively investigated by the luciferase-reporter assay. Vector intracellular trafficking is monitored in real time and in live cells by confocal microscopy. In particular, fluorescence resonant energy transfer (FRET) between suitably-labeled peptide and DNA modules was exploited to monitor complex disassembly during endocytosis, and this process is correlated to transfection timing and efficiency. We argue that these results can open the way to the rational design and application of CM_18_-Tat_11_–based systems for gene-delivery purposes.

## Introduction

Gene therapy is based on the principle that exogenous DNA can adjust the availability of deficient or somewhat altered gene products to normal physiological levels. Its success is largely dependent on the specific delivery-system properties since isolated nucleic acids are easily degraded in the external cell medium and are unable to penetrate biological membranes owing to their molecular weight and negative net charge. Several viral and non-viral approaches were investigated in the last decades [1,2], but they all suffer from severe drawbacks and limitations. For instance, viral vectors such as adenoviruses and retroviruses generally ensure efficient transfection [3] but their clinical use is hindered by several factors, including immune and inflammatory reactions, size limitation on cargo genes, and random integration into the host genome, in the case of retroviruses [4,5]. Physical methods such as hydroporation, electroporation, biolistic delivery (gene gun), or ultrasound are all used to deliver DNA across the plasma membrane and result in naked DNA being deposited into the cytoplasm, but can cause significant damage and raise a number of practical problems [6]. Design of non-viral vectors, such as liposomes [7], polymers [8], inorganic nanoparticles [9], and peptides [10] is gaining much attention as a potentially safe, low cost and multi-function option [5,6,11]. In particular, peptide-based materials offer the highly attractive feature of allowing the straightforward incorporation of the specific biological functionalities required for different biomedical applications (e.g. targeting). Importantly, peptides can be synthesized and characterized with well-established protocols, present relatively low cytotoxicity and immunogenicity, and often can be designed so that they degrade to naturally-occurring compounds in living systems. Concerning cell penetrating peptides (CPPs) in particular, several studies reported on their applicability to the delivery of DNA plasmids [12–18]. Unfortunately, regardless of the detailed peptide sequence and of its possible influence on the mechanism of entry, all reports show that peptide-DNA complexes are massively sequestered into vesicles (for a detail review see 19) and this hampers their transfection efficacy, when compared to well-established lipid-based systems [13,15,16]. To tackle this issue, we recently introduced the CM_18_-Tat_11_ chimeric peptide (Tat_11_: YGRKKRRQRRR, residues 47-57 of HIV-1 Tat protein; CM_18_: KWKLFKKIGAVLKVLTTG, residues 1-7 of Cecropin-A and 2-12 of Melittin antimicrobial peptides, respectively) [20]. The CM series attracted much interest since it comprises some of the smallest and most effective antimicrobial sequences with membrane-perturbing ability [21]. These hybrid peptides were constructed from various combinations of the hydrophilic N-terminal domain of Cecropin A with the hydrophobic N-terminal domain of Melittin, and tested on bacterial model systems [21]. We demonstrated that, upon fusion with Tat_11_, CM_18_ retains its structural and functional characteristics, i.e. it assumes the typical α-helical secondary structure in hydrophobic environments and preserves the ability to perturb membranes at the expected concentration; at the same time, Tat_11_ addition successfully increases CM_18_ cellular uptake and leads to effective internalization, with no detectable cytotoxic effects in the range of concentrations of interest [20]. When administered to cells, CM_18_-Tat_11_ combines two functionalities: efficient uptake and destabilization of endocytotic-vesicle membranes. This in turn effectively increases the intracellular availability of diverse membrane-impermeable molecules when co-administered, with no detectable cytotoxicity [20]. Here we exploit CM_18_-Tat_11_ physicochemical properties and intrinsic modularity to show its applicability as a delivery vector for plasmidic DNA, in this case following direct peptide-cargo conjugation. We demonstrate that CM_18_-Tat_11_/DNA complexes can be obtained spontaneously in solution, with controlled size, surface charge, and stability. Based on this, a selected candidate was identified, with the physicochemical properties better suited to yield a combination of high cellular uptake, low cytotoxicity, and efficient plasmid expression. Vector intracellular trafficking was monitored in real time and in live cells by confocal microscopy. In particular, fluorescence resonant energy transfer (FRET) between suitably-labeled DNA and peptide modules was exploited to monitor peptide-DNA disassembly during endocytosis, and correlate this process to transfection timing and efficiency. Finally, we propose a self-assembled CM_18_-Tat_11_/DNA delivery vector in which: i) the positively-charged arginine-rich Tat_11_ module binds non-covalently to the DNA phosphate groups and concomitantly provides high cellular uptake yields, while ii) the antimicrobial CM_18_ module exerts no interactions with the DNA, thus being available to promote successful plasmid intracellular delivery by its well-know membrane-disruptive properties. We believe this knowledge will provide useful guidelines for the rational application of CM_18_-Tat_11_ chimera for gene delivery purposes.

## Results

### CM_18_-Tat_11_ DNA binding ability: vector characterization

**Figure 1:**
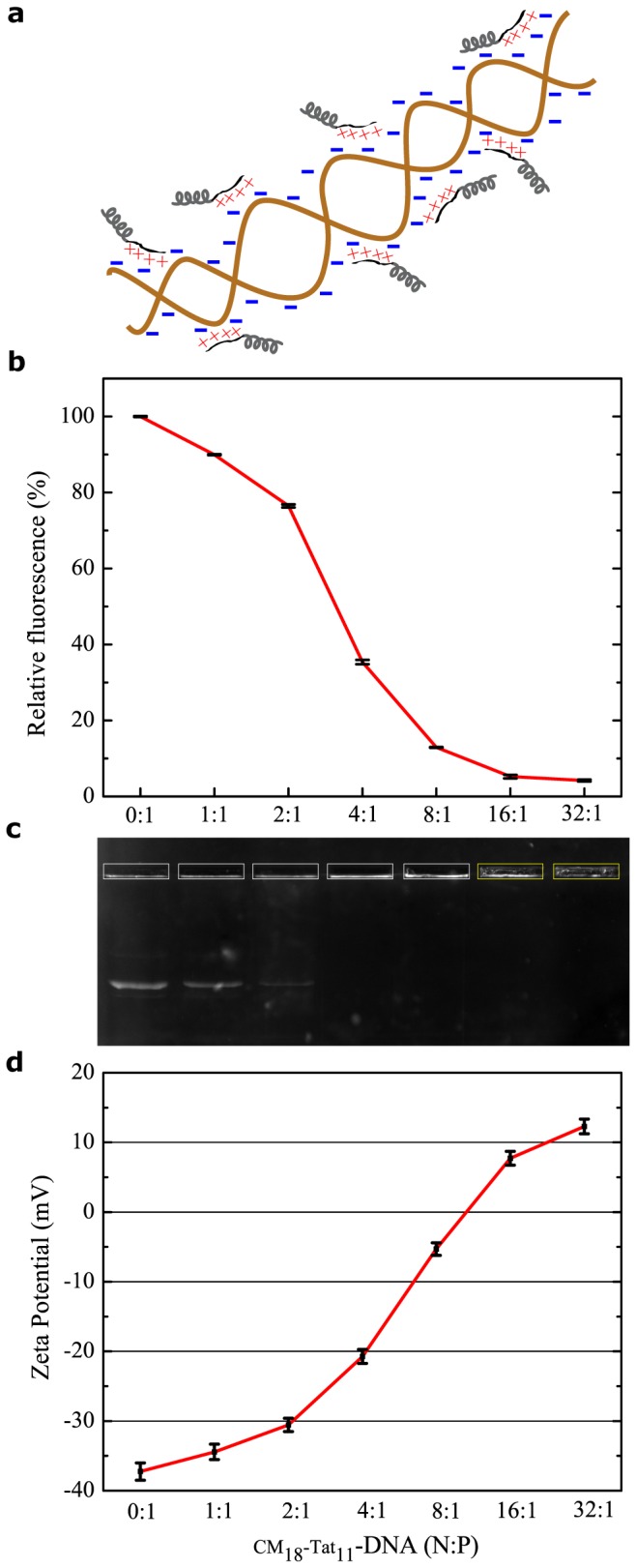
CM_18_-Tat_11_/DNA plasmid complex characterization. (**a**) Hypothetical ionic interaction model between DNA plasmid (brown fragment with blue negative charges) and CM_18_-Tat_11_ peptides (grey: CM_18_ and black: Tat_11_ with red positive charges). (**b**) CM_18_-Tat_11_ DNA condensation ability analyzed by EtBr exclusion assay. Results for complexes at N:P ratio from 0: 1 to 32: 1 are given as relative fluorescence and a value of 100% is attributed to the fluorescence of naked DNA with EtBr. The reported values represent the mean of three independent measurements, each performed in triplicate. (**c**) Stability of CM_18_-Tat_11_/plasmid DNA binary complex at N/P ratio as in (b) evaluated by agarose gel electrophoresis assay. Complexes are electrophoresed on a 0.8% (w/v) agarose gel with TBE running buffer at 80 V for 40 minutes. (**d**) ξ-potentials of CM_18_-Tat_11_/plasmid DNA binary complexes with N/P ratios as in (a) are measured at 25 °C by a Zetasizer Nano ZS90 instrument equipped with a red laser of wavelength 630 nm. Each sample is observed with 20 repeated measurements across 3 trials. Error bars in figures indicate standard deviations.

The ability of cationic peptides to bind and condense plasmidic DNA stems from ionic interactions between positively-charged amino acids and negatively-charged base pairs and can be exploited for the successful self-assembly of nanoparticles for gene-delivery [22–24] (see schematic representation in Figure 1a). Thus, to assess CM_18_-Tat_11_ suitability as a DNA carrier, we first performed a standard EtBr exclusion assay on peptide/DNA plasmid complexes at N:P ratios ranging from 0: 1 (isolated DNA) to 32:1. Figure 1b shows that CM_18_-Tat_11_ efficiently condenses the plasmid starting from an N:P ratio of 4:1 and is highly effective above 16:1 (complete exclusion of EtBr). Furthermore, by using an agarose-gel retardation assay (Figure 1c), we confirmed that CM_18_-Tat_11_/DNA complexes are progressively stabilized by increasing charge ratios: no bands corresponding to the isolated plasmid were detected in the gel at N:P ratios above 2:1. Please note that the diffuse signal detected in the loading-wells at N:P=16:1 and 32:1 also suggests an overall complex-charge inversion above the 8:1 threshold (yellow boxes in Figure 1c). In order to further investigate the physicochemical properties of peptide/DNA complexes, particle size and zeta potential were measured by dynamic light scattering. Table 1 shows that the particle-size trend nicely mirrors the DNA-protection effect as highlighted by the EtBr exclusion assay and that CM_18_-Tat_11_/DNA complexes appear progressively smaller in size for increasing N:P ratios. This trend probably stems from a ‘condensation effect’ brought by the electrostatics of the peptide-DNA interaction. Notably, the 32:1 complex does not further decrease in size consistently with the saturation of the DNA charges by the peptide positive residues. Additionally, we measured the peptide/DNA complex ξ-Potential: as reported in Figure 1d, nanoparticles formed at N:P ratios below 8:1 are in the -35 to -5 mV ξ-Potential range, while those formed at N:P ratios above 8:1 show ξ-Potential values above neutrality. It is worth mentioning that a diameter not exceeding 100-300 nm and a net positive nanoparticle charge are crucial for the vector to effectively bind to its negatively-charged cell-membrane counterparts and enter cells [1,19].

**Table 1:** Size distibution of CM_18_-Tat_1__1_/plasmid DNA binary complexes.

Charge ratio (N:P)	Z-Average diameter (nm)
1:1	135.0 ± 7.6
2:1	127.7 ± 3.3
4:1	113.9 ± 1.6
8:1	108.5 ± 1.9
16:1	101.8 ± 3.0
32:1	107.4 ± 2.8

Particle size of CM_18_-Tat_11_/plasmid DNA binary complexes with different N:P ratios are measured as explained in Figure 1d caption. Particle sizes are expressed as Z-average diameters (nm) and standard deviations are calculated from 20 repeated measurements across 3 trials.

### *In vitro* transfection efficiency

**Figure 2:**
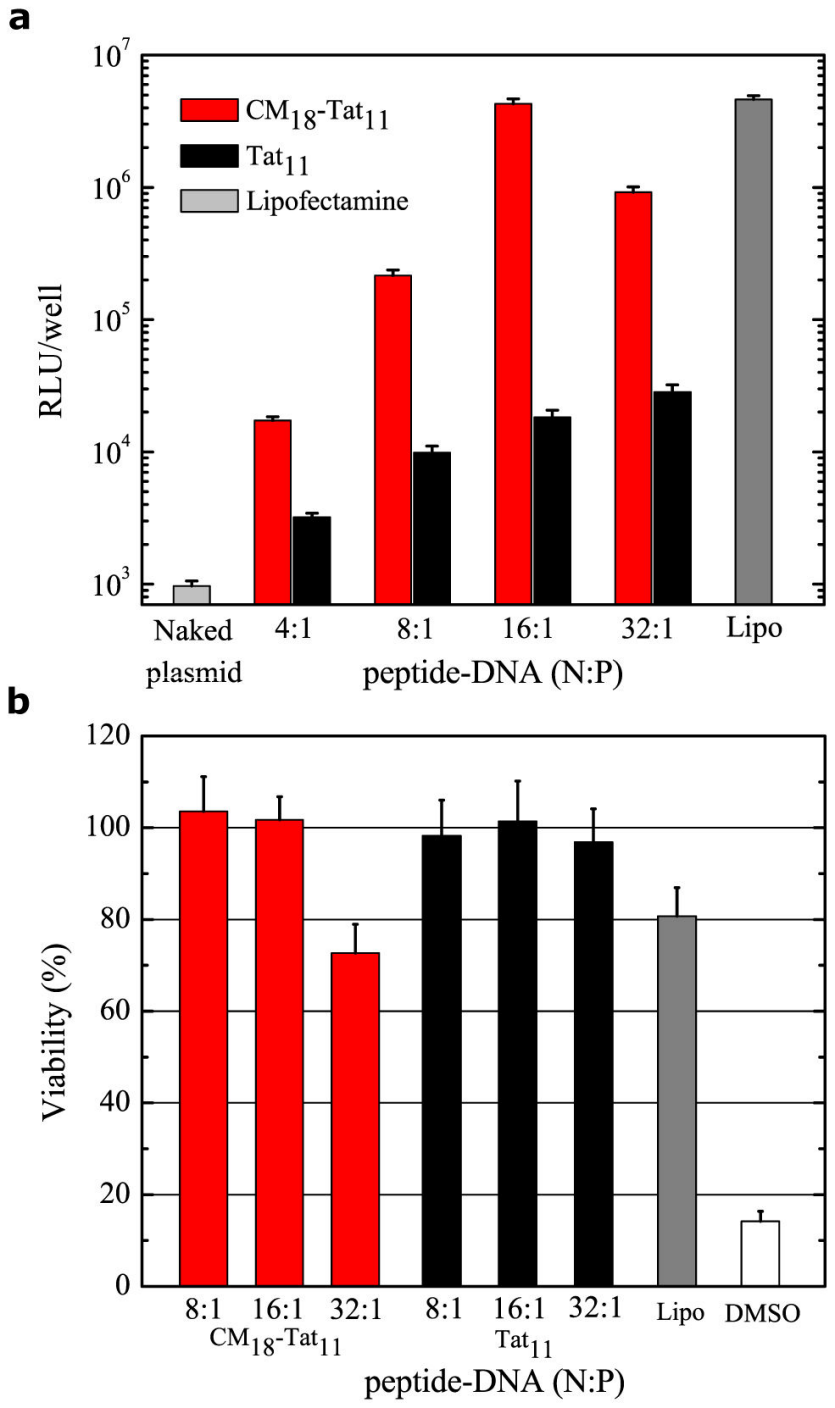
CM_18_-Tat_11_/DNA plasmid complex *in vitro* transfection efficiency and cytotoxicity. (**a**) Transgene expression is detected 24 hours after transfection by measuring luciferase activity from an aliquot of the HeLa cells external medium. Light grey column is the mean value obtained with naked DNA, dark grey column is for lipofectamine, while red and black column are respectively for CM_18_-Tat_11_ and Tat_11_ DNA complex at N:P ratio from 4:1 to 32:1. The reported RLU/well values represent the mean of three independent measurements, each performed in triplicate. (**b**) Wst-8 assay to evaluate cell metabolic activity. The wst-8 reagent was added for 2 hours and absorbance at 450 nm measured. Untreated cells are defined as 100% viable, while cells exposed to 20% dimethyl sulfoxide (white column) are used as positive control for decreased metabolic activity.

The transfection efficiency (TE) of CM_18_-Tat_11_/DNA complexes was measured in HeLa cells by a standard luciferase expression assay [20]. Based on the data reported above, only N:P ratios from 4:1 to 32:1 were considered eligible for further characterization. As shown in Figure 2a, luciferase gene expression increases by more than two orders magnitude from N:P=4:1 to N:P=16:1 (red columns), reaching a TE comparable with standard lipofection protocols (dark grey column). A further increase of N:P ratio to 32:1 does not lead to further TE improvement. As shown by the black columns in Figure 2a, isolated Tat_11_ yields a constant increase in TE for increasing N:P ratios, but at much lower levels compared to CM_18_-Tat_11_ chimera. This is not surprising [18] and can be linked to the massive trapping of the complex within endosomes characteristics of Tat_11_-mediated internalization [25,26]. Here we would like to stress that these Tat_11_ control experiments demonstrate the pivotal role of the CM_18_ module in the transfection process. We concluded this analysis by performing a complementary cytotoxicity assay (WST-8, see materials and methods) on the same cells. Figure 2b shows that classical lipofection yields a 20% viability reduction (dark grey column), while no significant cell toxicity was observed for Tat_11_/DNA and CM_18_-Tat_11_/DNA complexes (red and black columns), except for CM_18_-Tat_11_/DNA complex at a N:P ratio of 32:1, in line with the observed reduction in luciferase production (Figure 2a). The 32:1 complex corresponds to a CM_18_-Tat_11_ concentration of about 7 µM, a value very close to the hemolytic threshold previously calculated between 8 and 16 µM [20]. It is worth mentioning that similar measurements were also carried out with CHO cells, with analogous results (e.g. TE for the 16:1 complex is reported in Figure S1)

### Real-time imaging of CM_18_-Tat_11_/DNA uptake, integrity and transfection efficiency

**Figure 3:**
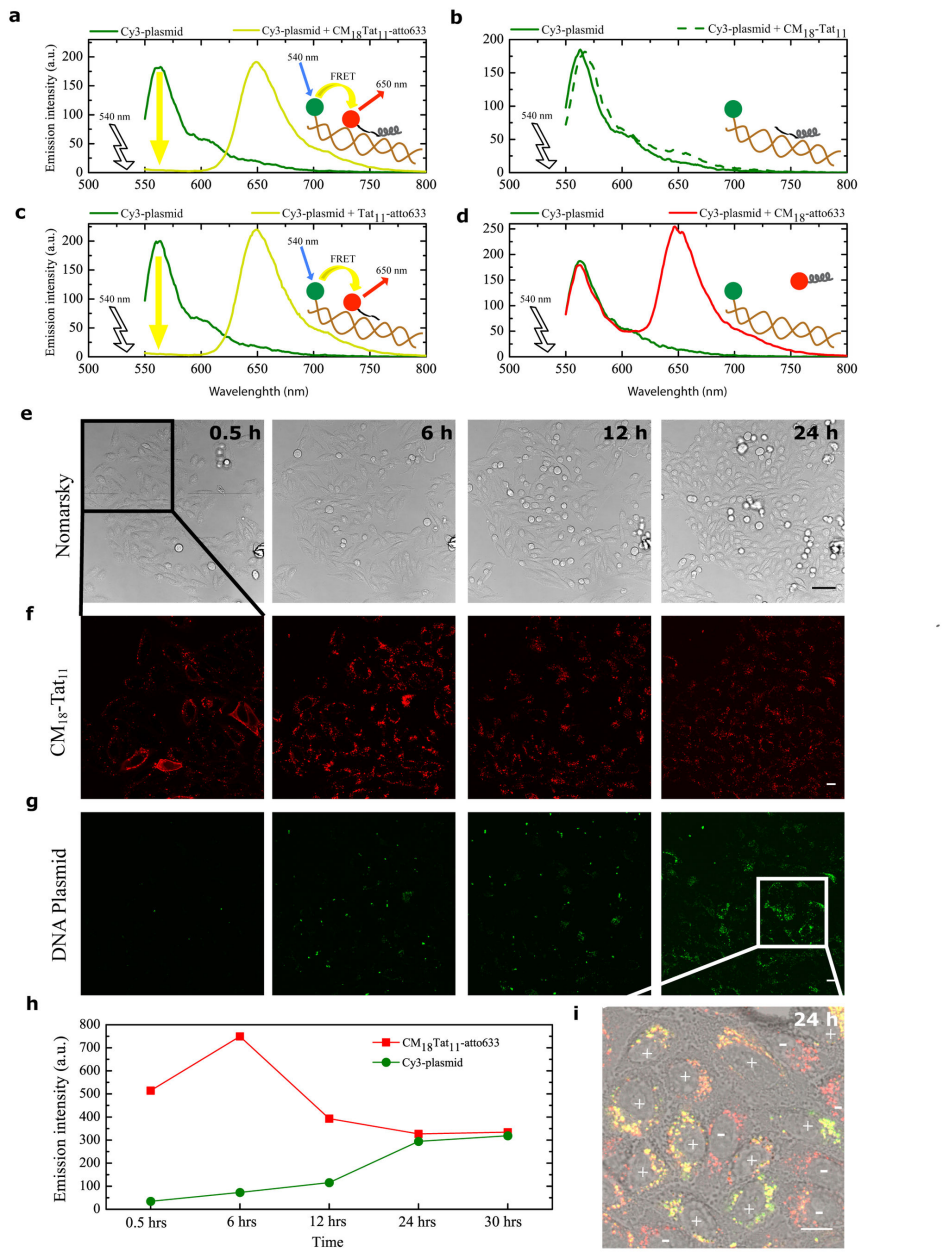
Spectroscopic properties and cell uptake dynamics of CM_18_-Tat_11_-atto633/DNA plasmid-Cy3 complexes. (**a**, **b**, **c**, **d**) Fluorescence spectra are recorded at 37 °C on a spectrofluorometer by exciting at 540 nm and collecting the fluorescence between 550 and 800 nm. First, a fluorescence emission measurement is performed on a PBS solution of Cy3-labeled DNA plasmid alone (solid green line in a, b, c, d). Then, atto633-labeled (dark yellow line in a) or unlabeled (dashed green line line in b) CM_18_-Tat_11_ or atto633-labeled Tat_11_ (dark yellow line in c) or atto633-labeled CM_18_ (red line in d) at N:P ratio 16:1 is added, and the emission spectrum recorded again (**e**) Nomarsky images of CLSM timelapse assay performed on cells incubated with CM_18_-Tat_11_-atto633/Cy3-plasmid DNA binary complexes at N:P ratio 16:1 applying the same treatments used for a typical transfection experiment. Scale bars: 50 µm. (**f**, **g**) Panels show the fluorescence signal distribution of CM_18_-Tat_11_-atto633 (f) and Cy3-plasmid (g) from the upper left quadrant of Figure 3e panels during the time-lapse. Scale bars: 10 µm. (**h**) Quantitative evaluation of CM_18_-Tat_11_-atto633 (red line) and Cy3-plasmid DNA (green line) signals during the time-lapse acquisition. (**i**) 24 hours CM_18_-Tat_11_-atto633 (f) and Cy3-plasmid (g) panels zoom merge. Scale bars: 10 µm.

**Figure 4:**
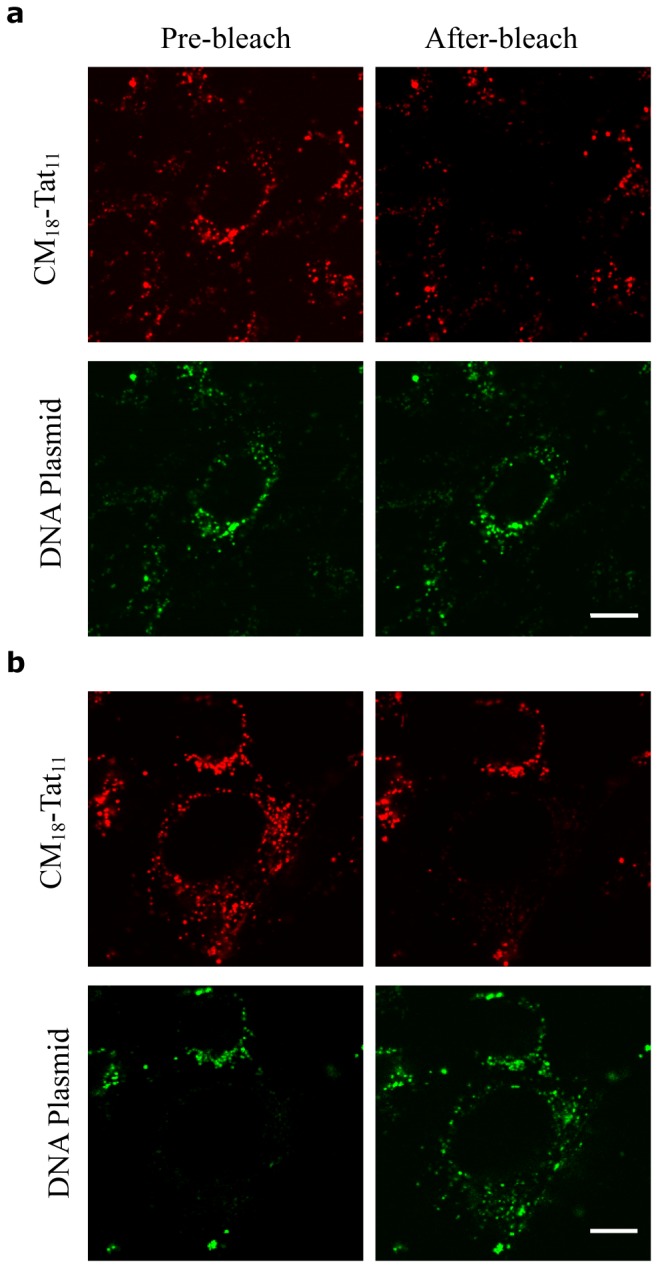
Acceptor photobleaching assay. (**a**, **b**) acceptor photobleaching experiments on putatively non-transfected (**a**) or trasfected (**b**) cells. For both conditions, the first row of images shows CM_18_-Tat_11_-atto633 cell signal before and after a scanning bleaching of the whole cell with a 633nm laser at full power, while the second row shows the same for Cy3-plasmid DNA signal. Scale bar: 10 µm.

**Figure 5:**
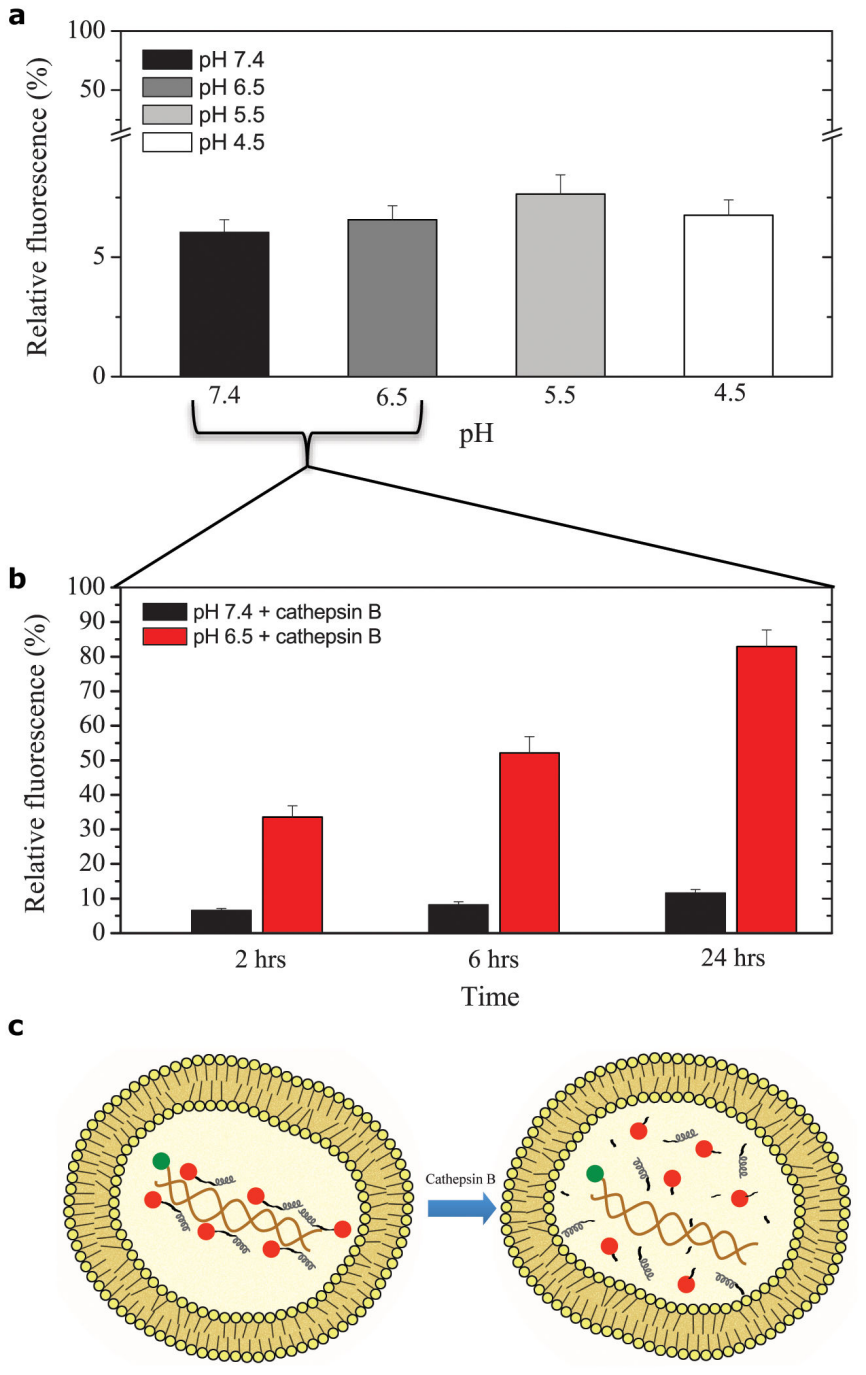
*In vitro* peptide/DNA complex stability evaluation in endosomal-like conditions. (**a**) EtBr exclusion assay performed as explained in caption 1b in PBS buffer at different pH: 7.4 (black column), 6.5 (dark grey), 5.5 (light grey), 4.5 (white). Y-axis break from 10% to 15% at 70% of the axis length. (**b**) EtBr exclusion assay 24 hours time-lapse performed at 37°C incubating CM_18_-Tat_11_/plasmid DNA binary complexes at pH=7.4 (black column) or pH=6.5 (red) with cathepsin B enzyme. (**c**) Graphical scheme of CM_18_-Tat_11_/plasmid DNA binary complexes disassembling evolution inside endosomal vesicle.

In order to gain better insight into the process of transfection by the peptide/DNA complexes used here, we set up a FRET-based real-time imaging experiment in living cells. Preliminary *in cuvette* spectroscopic characterization of the peptide/DNA complex was conducted by using a suitable pair of fluorophores (i.e. Cy3 as *donor*, labels the DNA and atto633 as *acceptor*, labels peptides; see materials and methods). As shown in Figure 3a, the characteristic donor-emission peak (560 nm; green curve) is fully quenched upon addition of the acceptor-labeled CM_18_-Tat_11_ (dark yellow curve). No quenching effect is detected upon addition of unlabeled CM_18_-Tat_11_ (Figure 3b solid-green and dashed-green curves): we can conclude that the observed quenching effect is indeed due to FRET (i.e. due to the close proximity of the two fluorophores within the peptide/DNA complex). Similar experiments were carried out with isolated Tat_11_ (Figure 3c) and CM_18_ (Figure 3d), as controls. The resulting spectra clearly indicate that the Tat_11_ module is the one responsible for DNA-binding (complete Cy3 quenching), while the CM_18_ module is likely unconstrained (unaltered Cy3 fluorescence spectrum upon acceptor addition). Finally, the atto633 fluorophore alone does not show any significant interaction with labeled plasmidic DNA, as reported in Figure S2. Based on this knowledge, we conclude that FRET is an effective probe to monitor complex integrity. Experimentally, we recorded Cy3 signal as a function of time: low-intensity signal implies stable complex, high-intensity signal implies free DNA plasmid. Thus, a confocal timelapse imaging experiment was performed on cells incubated with CM_18_-Tat_11_-atto633/plasmid DNA-Cy3 binary complexes at N:P ratio 16:1. The four panels in Figure 3e show the petri-dish region selected and imaged at low zoom for 24 hours. Note that in this time window cells are clearly replicating. The four panels in Figure 3f and 3g show, for a selected area of the field, the fluorescence-signal distribution of CM_18_-Tat_11_-atto633 and Cy3-DNA, respectively. Please note that, while CM_18_-Tat_11_-atto633 signal slightly decreases during transfection (red line in Figure 3h), the DNA-Cy3 signal grows (green line). We believe that former effect can be ascribed to the progressive metabolic degradation of the peptide module in the cells. After 72h incubation no detectable signal can be recorded (data not shown). On the contrary the increase in donor signal demonstrates that the peptide/DNA complex is disassembled within vesicles during transfection time. More in detail, up to 6h of internalization, the FRET effect is clearly visible (quenching of the *donor*, Figure 3g, 0.5h and 6h panels: Figure 3h): as stated above, this is proof of complex integrity. At 12h the *donor* emission becomes detectable and reaches its maximum after 24h of internalization (Figures 3g, 12h and 24h panels; Figure 3h). This loss of FRET indicates that the DNA-peptide complex is no more intact within vesicles. In this light, the overlay of the green (Cy3) and red (atto633) signals at 24h (see Figure 3i) can be used to identify the cells where complex disassembly is taking place (high donor, high acceptor, good colocalization, ‘+’ labels in Figure 3i) and distinguish them from those where the complex is likely still intact (low donor, high acceptor, poor colocalization, ‘-’ labels in the figure). If we assume that these two phenotypes correspond to transfected and non-transfected cells, respectively, we obtain a 70% TE estimate by simply counting the ‘positive’ cells at 24h, a level reminiscent of those typically obtained in transfection assays. In order to strengthen our overall interpretation of FRET experiments, we performed acceptor-photobleaching measurements at 24 hours on the two identified cell phenotypes (Figure 4). As shown in Figure 4a, selective photobleaching of the atto-633 acceptor signal on a putatively ‘non-transfected’ cell raises the donor fluorescence signal, thus showing that the cell is (still) loaded with intact peptide/DNA complexes. On the other hand, photobleaching of the acceptor in a putatively ‘transfected’ cell does not alter the intensity of the Cy3 donor signal, confirming that the two fluorophores are not in close proximity: this in turn may result either from simple detachment of the two intact modules (DNA and peptide) or from metabolic degradation of one or both modules. It is worth highlighting that the latter possibility is consistent with the clear colocalization observed after 12h between the DNA-CM_18_-Tat_11_ complex and a marker of the lysosome, the subcellular compartment dedicated to metabolic degradation (Figure S3). In the attempt to further identify the specific metabolic processes responsible for such a regulation of peptide/DNA complex integrity during vesicular trafficking, we turned to *in cuvette* measurements and tested complex integrity in time by the EtBr exclusion-assay under selected conditions. As shown in Figure 5a, the lowering of pH from 7.4 to 4.5 (grey scale), which typically occurs within vesicles, does not affect the complex stability. In keeping with the final destination of the internalized complexes into lysosomes (see above), addition of the ubiquitous Cathepsin-B lysosomial enzyme induces almost complete complex disassembly within 24h incubation (with kinetics reminiscent of the FRET loss in cells), but only at pH below 6.5, as expected for this enzyme (Figure 5b). Cathepsin-B cutting site is the peptide bond between adjacent arginine residues: the exclusive localization of arginine amino acid couples in the Tat_11_ sequence makes this result an additional clue for the crucial role of this module in complex formation (i.e. DNA binding).

## Discussion

An ideal cell penetrating peptide for the successful delivery of DNA into cells should perform at least three different tasks: (1) it should tightly bind to the DNA, thus forming a vector molecule of controlled physicochemical properties capable of protecting the plasmid from early degradation in the extracellular medium; (2) it should favor high-yield, non-toxic cellular uptake of the DNA-cargo moiety; (3) it should provide an efficient intracellular route for cargo endosomal escape, thus allowing for an efficient transfection efficiency. We recently showed that the last two properties are potentially offered by a new class of chimeric peptides where the highly-efficient, non-toxic uptake of classical CPPs (i.e. Tat_11_) is combined to the membrane-disruption ability of a linear cationic α-helical antimicrobial peptide (i.e. CM_18_). In particular, the CM_18_-Tat_11_ chimera was shown to possess the correct balance between ‘peptide active concentration’ (i.e. the concentration needed to perturb membrane integrity) and the specific cellular location where this concentration is achieved (the endosome) thereby promoting efficient intracellular delivery of a variety of biomolecules, including DNA [20]. However, this property was proved when cargo molecules were co-administered and actually randomly encountered by the membrane-disruptive peptide in a subset of vesicles during their independent intracellular trafficking. The ‘colocalization’ prerequisite can severely hinder effective (and controlled) delivery of plasmids into cells. In order to tackle this issue and satisfy criterion (1) above, in this article we tested the ability of the CM_18_-Tat_11_ chimera to efficiently and safely deliver plasmidic DNA into living cells following its *conjugation* to the DNA cargo. We demonstrated that the CM_18_-Tat_11_ possesses intrinsic DNA binding properties: upon mixing the former with the plasmid, they spontaneously generate a mono-disperse solution of nanoparticles, with controlled physicochemical characteristics, in term of size, surface charge, and stability. By FRET-based spectrofluorimetric assays we showed that DNA-binding properties are associated to the Tat_11_ module alone, in keeping with previous observations by some of us [27]. The α-helical CM_18_ module is dispensed from intra-molecular interactions and can exert its membrane-disruption functions. By the well-established luciferase assay, we demonstrated that the 16:1 vector molecule does possess the ability to enter cells and promote plasmid expression at levels comparable to those achievable by widely-used lipid-based systems. In this respect, it is worth mentioning that analogous peptide-based vectors for *in vitro* transfection usually do not reach the efficiency level of lipid standards, as detailed in several reports [13,16–16]. This can be explained by the massive peptide/DNA complex entrapment within endocytic vesicles, a major limiting factor that is successfully addressed here by the endosomolitic activity of the CM_18_ module. Like all the other peptide-based strategies, CM_18_-Tat_11_ ensures high transfection with no associated toxicity. The latter result is important since, although being golden standards for transfection assays, lipid formulations typically elicit oxygen radical-mediated cell toxicity in both immortalized and primary cells [13,28] (e.g. 20% reduction in cell viability measured here). As demonstrated elsewhere [13], being non-toxic is a key prerequisite for a successful application of the vector *in vivo*. In order to get further insight into the detailed mechanisms driving the transfection process, we monitored the intracellular trafficking of fluorescently-labeled peptide-DNA complexes by confocal imaging. In particular, we used FRET measurements to monitor the peptide-DNA complex integrity in real time at cell level. By this approach we demonstrated that the complex progressively disassembles during its vesicular trafficking, with the DNA becoming virtually completely naked (and therefore competent for transfection) within 24h of treatment. Interestingly, the measured timing of complex disassembly mirrors that of actual transcription and translation of the luciferase reporter gene. Based on *in cuvette* measurements, we argued that pH-activated intra-vesicular enzymatic activity (e.g. by cathepsin B within lysosomes) may affect peptide integrity, and consequently lead to its release from DNA within vesicles (see schematic representation in Figure 5c). Overall our data support a model in which: a stable, self-assembled, ~100 nm-sized peptide/DNA complex enters cells by endocytosis. During the physiological vesicular trafficking, the complex reaches the lysosomial compartment where at least two mechanisms jointly favor DNA release: i) the Tat_11_ module is enzymatically degraded, thus releasing both the plasmid and the CM_18_ module (FRET and cathepsin B data); ii) once at its critical membrane-perturbing concentration, the CM_18_ module destabilizes bilayer integrity and promotes DNA release. It is worth underlining that the release of such large molecules (hydrodynamic radius larger than 100 nm) can be accounted for only by the detergent-like destabilization of the vesicular membrane postulated under the ‘carpet model’ for linear α-helical antimicrobial peptides [21], as opposed to the ‘barrel-stave’ and ‘toroidal’ pore models, in keeping with our previous data [21]. Somewhat contradictorily, our data show that the expression of luciferase occurs together with a massive entrapment of (naked) DNA molecules into vesicles. No reports thus far have fully clarified the mechanism of transport of plasmidic DNA from vesicles to the nucleus, but analogously to what shown here, the expression of a reporter gene is used as proof of the actual nuclear localization of the plasmid. Consistently with our results other authors showed that when a fluorescently-labeled variant of DNA is used, no detectable amount of fluorescence is observed in the nucleus, even when high-delivery lipid vectors are used [29–31]. We are led to deduce that only few DNA molecules (below the detection limit) successfully escape from vesicles and gain access to the nucleus. This interpretation is supported by several studies on lipofection showing that a large fraction of the input DNA never reaches the nucleus [32–34]. Interestingly, two studies showed that between 30 and 100 times more plasmid microinjected into the cytoplasm is necessary to give equivalent levels of gene expression compared injection directly into the nucleus [35,36]. Indeed DNA trafficking through the cytoplasm is inefficient and degradation likely plays a major role in limiting the amount of DNA that can reach the nucleus. It was estimated that the half-life of naked plasmid DNA in the cytoplasm of cells ranges between 50 minutes and 5 hours, so that if a half-life of 3 hours is assumed, during a typical 24 hour transfection experiment, less than 0.4% of the input DNA would remain by 24 hours [33,34,37–41]. In conclusion, we believe that these results on CM_18_-Tat_11_-mediated DNA transfection in live cells can represent a useful basis for the rational design of optimized modular peptide-based carriers for gene-therapy applications.

## Methods

### Cell culture, peptides and DNA plasmids

HeLa and Chinese Hamster Ovary (CHO-K1) cells were purchased from ATCC and cultured following manufacturer’s instructions. Cells were maintained at 37°C in a humidified 5% CO_2_ atmosphere. All peptides were prepared by solid-phase synthesis using Fmoc chemistry on an automatic Liberty-12-Channel Automated Peptide Synthesizer. Crude peptides were purified by RP-HPLC on a Jupiter 4m Proteo 90 A column (250 × 10 mm; Phenomenex). The HPLC analysis and purification was performed on a Dionex Ultimate 3000 PLC system with autosampler. The correct purified product was confirmed by electrospray mass spectroscopy. Purity was >95% as determined by analytical high-performance liquid chromatography (HPLC). The Cysteine residue added to the C-terminus of each peptide provided a sulfhydryl group for further ligation to the atto-633-maleimide fluorophore. The labeling of purified peptides was performed by incubating for 3 h with a 3-fold molar excess of atto-633-maleimide (ATTO-TEC GmbH, Germany), 150 mM PBS buffer, TCEP, at pH 7.4. Finally, atto-633-labeled peptides were purified by HPLC (see above) and then lyophilized overnight. The molecular weight of all conjugated peptides was confirmed by electrospray mass spectroscopy and the concentration of each peptide stock solution was verified by UV–vis absorbance. The ESI-MS spectra of the peptides were obtained with an API3200QTRAP a Hybrid Triple Quadrupole/Linear Ion Trap (ABSciex, Foster City, California, USA). Peptides were stored at -80 °C. pCMV-GLuc 2 control plasmid (5.7 kb, 3700 kDa; New England Biolabs, Ipswich, MA) was used as a reporter gene. pCMV-GLuc 2 control plasmid was first transformed in One Shot TOP 10 chemically competent Escherichia coli and then was amplified in Luria Bertani broth media at 37 °C overnight. After that, a Plasmid Maxi Kit (Qiagen, Valencia, CA) was used for purification of the plasmid. The purified plasmid DNA was dissolved in water at 1 μg/μl concentration and stored at -20 °C before use. Label IT® Cy3 plasmid delivery control (2.7 kb, 1730 kDa; Mirus Bio Corporation, Madison, WI) was used for confocal fluorescence microscopy experiments and agarose gel electrophoresis.

### Transfection vectors

Peptide/Plasmidic DNA binary complexes were prepared as follows: 1 μg of plasmidic DNA was dissolved in 200 μl of PBS solution and different volumes of the peptide solution were added to obtain the desired N:P molar ratio. The mixture was then incubated at room temperature for 30 minutes. Finally, the transfection medium was added till 1 ml total volume (i.e. in CM_18_-Tat_11_:DNA complex 4:1, concentrations are 0.8 μM peptide and 0.3 nM DNA).

### *In vitro* DNA transfection

Cells were plated in a 96-well plate at a density of 8 × 10^4^ cells/ml, cultivated in growth medium with 10% FBS. One hundred μl of medium was added to each well. After 24 hours growth medium in each well was replaced with 100μl transfection medium containing peptide/plasmid DNA binary complexes. Incubation with the cells lasted 6 hours at 37 °C. Then, 200 μl growth medium replaced the transfection medium and cells were cultured for 24 hours at 37 °C in 5% (v/v) CO_2_ after transfection. All transfection assays were carried out three times and each in sestuplicate simultaneously. For control lipid transfection Lipofectamine (Invitrogen, Carlsbad, CA) was used according to the manufacturer’s instruction. For confocal live scanning microscopy (CLSM) cells were plated onto 35 mm glass-bottom petri dishes (WillCo-dish GWSt-3522) the day before the experiment to reach 70% confluence. After 24 hours growth cells medium was replaced with 1 ml transfection medium containing CM_18_-Tat_11_-atto633/Cy3-plasmid DNA binary complexes at N:P ratio 16/1 prepared as explained in section 2.2. Incubation with the cells lasted 6 hours at 37 °C. Then we replaced transfection medium with growth medium.

### Detection of transgene expression

Transgene expression was detected 24 hours after transfection. The luciferase activity was measured from an aliquot of the external medium by using an assay kit from New England Biolabs and an injector-equipped Veritas microplate luminometer (Turner BioSystems, Sunnyvale, CA). The RLU/well unit was used to present the most accurate expression level in this experimental system.

### Cytotoxicity evaluation

To test the effect of peptide/plasmid DNA binary complexes treatment on cell metabolism we used the Wst-8 (Water soluble tetrazolium) assay (Sigma-Aldrich, Buchs, Switzerland) on the same cell employed for luciferase assay. In detail after luciferase sampling cells were exposed to the Wst-8 reagent according to the manufacturer’s protocol. Absorbance at 450 nm was measured 2 hours later on a Synergy HT multiplate reader (Bio Tek instruments, Winooski, VT). Untreated cells were defined as 100% viable, while cells exposed to 20% dimethyl sulfoxide (DMSO) for 1 hour were used as positive control.

### Ethidium bromide (EtBr) exclusion assay

DNA condensation was analyzed using an EtBr (Sigma, Taufkirchen, Germany) exclusion assay. Briefly, complexes were formed as described above in section 2.2. After 1 hour incubation, each sample was transferred into a black 96-well plate. Thereafter, 3.2 μl of EtBr solution was added to give a final EtBr concentration of 400nmol/l. After 10 minutes, fluorescence was measured on a Synergy HT multiplate reader (Bio Tek instruments) at λ_ex_ = 525nm and λ_em_ = 620nm. Results are given as relative fluorescence and a value of 100% is attributed to the fluorescence of naked DNA with EtBr. The same assay was repeated in a 24 hours time-lapse incubating 1: 16 N:P ratio CM_18_-Tat_11_/plasmid DNA binary complexes in different solutions at 37°C: PBS buffer at pH=7.4 ± cathepsin B enzyme (Sigma-Aldrich, 10mU), PBS buffer at pH=6.5 (by addition of HCl) ± cathepsin B enzyme, PBS buffer at pH=5.5 or PBS buffer at pH=4.5.

### Agarose gel electrophoresis

The stabilities of CM_18_-Tat_11_/plasmid DNA binary complexes with different N:P ratio were evaluated by agarose gel electrophoresis assay. In brief 2 μL DNA Cy3 plasmid solution was mixed with the vector solutions at different N:P ratios in 200 μl PBS as final volume. The system incubated at 37 °C for 30 minutes. After complexes (50 μl per well) were electrophoresed on the 0.8% (w/v) agarose gel containing with TBE running buffer at 80 V for 40 minutes, an ImageQuant LAS 4000 biomolecular imager (GE Healthcare, Madison, WI) was used for the visualization of DNA position inside the gel. Naked DNA Cy3 plasmid was used as control.

### Particle size and ξ-potential measurements

Particle size and ξ-potentials of CM_18_-Tat_11_/plasmid DNA binary complexes with different N:P ratios were measured at 25 °C by a Zetasizer Nano ZS90 (Malvern Instruments Ltd., UK) instrument equipped with a red laser of wavelength 630 nm. All the complexes were prepared using pCMV-GLuc 2 control plasmid in a total volume of 1 ml of PBS. Each sample was observed with 20 repeated measurements across 3 trials. Error bars in figures indicate standard deviations.

### *In vitro* spectroscopic Förster Resonance Energy Transfer (FRET) measurements

Fluorescence spectra were recorded at 37 °C on a Cary Eclipse spectrofluorometer (Varian) by setting the excitation and emission monochromator slits both to 5 nm; the scanning speed was set to 120 nm/min, and data resolution to 1 nm. Emission spectra were recorded by exciting at 540 nm and collecting the fluorescence between 550 and 800 nm. First, a fluorescence emission measurement was performed on a PBS solution of Cy3-labeled DNA plasmid alone. Then, atto633-labeled peptide at N:P ratio 16:1 was added, and the 550-800-nm emission spectrum recorded. FRET can be unequivocally measured by the extent of donor-emission quenching, as this depends only on donor-acceptor proximity in the complex (addition of unlabeled peptide does not affect donor emission). On the contrary, acceptor fluorescence does not only depend on the extent of its sensitized emission (FRET) but also on the sum of acceptor cross-excitation at 540 and acceptor quenching due to DNA binding (unlabeled plasmid is able to quench atto633-peptide fluorescence; data not shown).

### Confocal laser scanning microscopy (CLSM) and image analysis

CLSM experiments were performed using a Leica TCS SP5 inverted confocal microscope (Leica Microsystems AG, Wetzlar, Germany), interfaced with a He–Ne laser for excitation at 561 and 633 nm. Glass-bottom petri dishes containing transfected cells were mounted in a thermostated chamber (Leica Microsystems) and viewed with a 40×1.25 numerical aperture oil immersion objective (Leica Microsystems). Live cell imaging was always performed at 37 °C. Emission was monitored by means of the Acousto-Optical Beam Splitter (AOBS)-based built-in detectors of the confocal microscope. The following collection ranges were adopted: 460-530 nm (Lysosensor), 570-610 nm (Cy3), and 650-750 nm (atto-633). For FRET imaging, we did not acquire the sensitized-emission channel (excitation: 543 nm, collection 650-750 nm) because it is not ideal for monitoring the complex stability due to the co-presence of some external contributions, as atto-633 own excitation at 540 nm and DNA quenching effect (data not shown). In a typical two-channel experiment images were collected in sequential mode to eliminate emission cross talk or bleed through between the various dyes. Acceptor photobleaching experiments for the analysis of complex integrity inside cell vesicles started with an eight-time line-averaged image of the cell followed by a scanning bleaching of the whole cell with a 633nm laser at full power for the minimum time required to photobleach the vesicular fluorescence signal in the second channel. After the bleaching we took a new eight-time line-averaged image of the cell for both channels. All data collected were analyzed by ImageJ software version 1.37 (NIH Image; http://rsbweb.nih.gov/ij/).

## Supporting Information

Figure S1 CM_18_-Tat_11_/DNA *in vitro* transfection efficiency in CHO cells. Transgene expression is detected 24 hours after transfection by measuring luciferase activity from an aliquot of the CHO cells external medium. Light grey column is the mean value obtained with naked DNA, dark grey column is for lipofectamine, while red column is for CM_18_-Tat_11_/DNA complex at N:P ratio 16:1. The reported RLU/well values represent the mean of three independent measurements, each performed in triplicate.
(TIFF)

Figure S2 Spectroscopic properties of Cy3-DNA/atto633 complex. Fluorescence spectra are recorded at 37 °C with a spectrofluorometer by exciting at 540 nm and collecting the fluorescence between 550 and 800 nm. First, a fluorescence emission measurement is performed on a PBS solution of Cy3-labeled DNA plasmid alone (solid green line). Then, isolated atto633 (solid red line) at N:P ratio 16:1 is added, and the emission spectrum recorded again. No donor quenching is detected.
(TIF)

Figure S3 Intracellular final fate of DNA/peptide complex in HeLa cells. Colocalization of Lysosensor signal (lysosome marker, green) with atto633-CM_18_-Tat_11_/DNA 16:1 complex signal (red) after 12 h of treatment. The overlay (yellow) reveals that the complex is completely delivered to the lysosomal compartment.
(TIF)

## References

[1] Luo D, Saltzman WM (2000) Synthetic DNA delivery systems. Nat Biotechnol 18: 33-37. doi:10.1038/71889. PubMed: 10625387. 10625387 10.1038/71889

[2] Kay MA, Glorioso JC, Naldini L (2001) Viral vectors for gene therapy: the art of turning infectious agents into vehicles of therapeutics. Nat Med 7: 33-40. doi:10.1038/83324. PubMed: 11135613. 11135613 10.1038/83324

[3] Warnock JN, Daigre C, Al-Rubeai M (2011) Introduction to viral vectors. Methods Mol Biol 737: 1-25. doi:10.1007/978-1-61779-095-9_1. PubMed: 21590391. 21590391 10.1007/978-1-61779-095-9_1

[4] Thomas CE, Ehrhardt A, Kay MA (2003) Progress and problems with the use of viral vectors for gene therapy. Nat Rev Genet 4: 346-358. doi:10.1038/nrg1066. PubMed: 12728277. 12728277 10.1038/nrg1066

[5] Viola JR, El-Andaloussi S, Oprea II, Smith CI (2010) Non-viral nanovectors for gene delivery: factors that govern successful therapeutics. Expert Opin Drug Deliv 7: 721-735. doi:10.1517/17425241003716810. PubMed: 20367531. 20367531 10.1517/17425241003716810

[6] Jo J, Tabata Y (2008) Non-viral gene transfection technologies for genetic engineering of stem cells. Eur J Pharm Biopharm 68: 90-104. doi:10.1016/j.ejpb.2007.04.021. PubMed: 17870447. 17870447 10.1016/j.ejpb.2007.04.021

[7] Li S, Huang L (2000) Nonviral gene therapy: promises and challenges. Gene Ther 7: 31-34. doi:10.1038/sj.gt.3301110. PubMed: 10680013. 10680013 10.1038/sj.gt.3301110

[8] Pack DW, Hoffman AS, Pun S, Stayton PS (2005) Design and development of polymers for gene delivery. Nat Rev Drug Discov 4: 581-593. doi:10.1038/nrd1775. PubMed: 16052241. 16052241 10.1038/nrd1775

[9] Petros RA, DeSimone JM (2010) Strategies in the design of nanoparticles for therapeutic applications. Nat Rev Drug Discov 9: 615-627. doi:10.1038/nrd2591. PubMed: 20616808. 20616808 10.1038/nrd2591

[10] Bolhassani A (2011) Potential efficacy of cell-penetrating peptides for nucleic acid and drug delivery in cancer. Biochim Biophys Acta 1816: 232-246. PubMed: 21840374. 21840374 10.1016/j.bbcan.2011.07.006

[11] Glover DJ, Lipps HJ, Jans DA (2005) Towards safe, non-viral therapeutic gene expression in humans. Nat Rev Genet 6: 299-310. doi:10.1038/nrg1577. PubMed: 15761468. 15761468 10.1038/nrg1577

[12] Nakase I, Akita H, Kogure K, Gräslund A, Langel U et al. (2012) Efficient intracellular delivery of nucleic acid pharmaceuticals using cell-penetrating peptides. Acc Chem Res 45: 1132-1139. doi:10.1021/ar200256e. PubMed: 22208383. 22208383 10.1021/ar200256e

[13] Lehto T, Simonson OE, Mäger I, Ezzat K, Sork H et al. (2011) A peptide-based vector for efficient gene transfer in vitro and in vivo. Mol Ther 19: 1457-1467. doi:10.1038/mt.2011.10. PubMed: 21343913. 21343913 10.1038/mt.2011.10 PMC3149163

[14] Ignatovich IA, Dizhe EB, Pavlotskaya AV, Akifiev BN, Burov SV et al. (2003) Complexes of plasmid DNA with basic domain 47-57 of the HIV-1 Tat protein are transferred to mammalian cells by endocytosis-mediated pathways. J Biol Chem 278: 42625-42636. doi:10.1074/jbc.M301431200. PubMed: 12882958. 12882958 10.1074/jbc.M301431200

[15] Liu Z, Li M, Cui D, Fei J (2005) Macro-branched cell-penetrating peptide design for gene delivery. J Control Release 102: 699-710. doi:10.1016/j.jconrel.2004.10.013. PubMed: 15681091. 15681091 10.1016/j.jconrel.2004.10.013

[16] Mäe M, El Andaloussi S, Lundin P, Oskolkov N, Johansson HJ et al. (2008) A stearylated CPP for delivery of splice correcting oligonucleotides using a non-covalent co-incubation strategy. J Control Release 134: 221-227. PubMed: 19105971. 19105971 10.1016/j.jconrel.2008.11.025

[17] Liu BR, Lin MD, Chiang HJ, Lee HJ (2012) Arginine-rich cell-penetrating peptides deliver gene into living human cells. Gene 505: 37-45. doi:10.1016/j.gene.2012.05.053. PubMed: 22669044. 22669044 10.1016/j.gene.2012.05.053

[18] Yi WJ, Yang J, Li C, Wang HY, Liu CW et al. (2012) Enhanced nuclear import and transfection efficiency of TAT peptide-based gene delivery systems modified by additional nuclear localization signals. Bioconjug Chem 23: 125-134. doi:10.1021/bc2005472. PubMed: 22148643. 22148643 10.1021/bc2005472

[19] Hoyer J, Neundorf I (2012) Peptide vectors for the nonviral delivery of nucleic acids. Acc Chem Res 45: 1048-1056. doi:10.1021/ar2002304. PubMed: 22455499. 22455499 10.1021/ar2002304

[20] Salomone F, Cardarelli F, Di Luca M, Boccardi C, Nifosì R et al. (2012) A novel chimeric cell-penetrating peptide with membrane-disruptive properties for efficient endosomal escape. J Control Release 163: 293-303. doi:10.1016/j.jconrel.2012.09.019. PubMed: 23041543. 23041543 10.1016/j.jconrel.2012.09.019

[21] Sato H, Feix JB (2006) Peptide-membrane interactions and mechanisms of membrane destruction by amphipathic alpha-helical antimicrobial peptides. Biochim Biophys Acta 1758: 1245-1256. doi:10.1016/j.bbamem.2006.02.021. PubMed: 16697975. 16697975 10.1016/j.bbamem.2006.02.021

[22] Meade BR, Dowdy SF (2008) Enhancing the cellular uptake of siRNA duplexes following noncovalent packaging with protein transduction domain peptides. Adv Drug Deliv Rev 60: 530-536. doi:10.1016/j.addr.2007.10.004. PubMed: 18155315. 18155315 10.1016/j.addr.2007.10.004 PMC2293332

[23] Deshayes S, Morris M, Heitz F, Divita G (2008) Delivery of proteins and nucleic acids using a non-covalent peptide-based strategy. Adv Drug Deliv Rev 60: 537-547. doi:10.1016/j.addr.2007.09.005. PubMed: 18037526. 18037526 10.1016/j.addr.2007.09.005

[24] Wender PA, Mitchell DJ, Pattabiraman K, Pelkey ET, Steinman L et al. (2000) The design, synthesis, and evaluation of molecules that enable or enhance cellular uptake: peptoid molecular transporters. Proc Natl Acad Sci U S A 97: 13003-13008. doi:10.1073/pnas.97.24.13003. PubMed: 11087855. 11087855 10.1073/pnas.97.24.13003 PMC27168

[25] Lundin P, Johansson H, Guterstam P, Holm T, Hansen M et al. (2008) Distinct uptake routes of cell-penetrating peptide conjugates. Bioconjug Chem 19: 2535-2542. doi:10.1021/bc800212j. PubMed: 19012426. 19012426 10.1021/bc800212j

[26] Erazo-Oliveiras A, Muthukrishnan N, Baker R, Wang T, Pellois J (2012) Improving the endosomal escape of cell-penetrating peptides and their cargos: strategies and challenges. Pharmaceuticals 5: 1177-1209. 24223492 10.3390/ph5111177 PMC3816665

[27] Cardarelli F, Serresi M, Bizzarri R, Beltram F (2008) Tuning the transport properties of HIV-1 Tat arginine-rich motif in living cells. Traffic 9: 528-539. doi:10.1111/j.1600-0854.2007.00696.x. PubMed: 18182009. 18182009 10.1111/j.1600-0854.2007.00696.x

[28] Gooding M, Browne LP, Quinteiro FM, Selwood DL (2012) siRNA delivery: from lipids to cell-penetrating peptides and their mimics. Chem Biol Drugs Des 80: 787-809. doi:10.1111/cbdd.12052. PubMed: 22974319. 10.1111/cbdd.12052 22974319

[29] Zabner J, Fasbender AJ, Moninger T, Poellinger KA, Welsh MJ (1995) Cellular and molecular barriers to gene transfer by a cationic lipid. J Biol Chem 270: 18997-19007. doi:10.1074/jbc.270.32.18997. PubMed: 7642560. 7642560 10.1074/jbc.270.32.18997

[30] Cardarelli F, Pozzi D, Bifone A, Marchini C, Caracciolo G (2012) Cholesterol-dependent macropinocytosis and endosomal escape control the transfection efficiency of lipoplexes in CHO living cells. Mol Pharm 9: 334-340. doi:10.1021/mp200374e. PubMed: 22196199. 22196199 10.1021/mp200374e

[31] Pozzi D, Marchini C, Cardarelli F, Amenitsch H, Garulli C et al. (2012) Transfection efficiency boost of cholesterol-containing lipoplexes. Biochim Biophys Acta 1818: 2335-2343. doi:10.1016/j.bbamem.2012.05.017. PubMed: 22627109. 22627109 10.1016/j.bbamem.2012.05.017

[32] Coonrod A, Li FQ, Horwitz M (1997) On the mechanism of DNA transfection: efficient gene transfer without viruses. Gene Ther 4: 1313-1321. doi:10.1038/sj.gt.3300536. PubMed: 9472555. 9472555 10.1038/sj.gt.3300536

[33] James MB, Giorgio TD (2000) Nuclear-associated plasmid, but not cell-associated plasmid, is correlated with transgene expression in cultured mammalian cells. Mol Ther 1: 339-346. doi:10.1006/mthe.2000.0054. PubMed: 10933952. 10933952 10.1006/mthe.2000.0054

[34] Tseng WC, Haselton FR, Giorgio TD (1997) Transfection by cationic liposomes using simultaneous single cell measurements of plasmid delivery and transgene expression. J Biol Chem 272: 25641-25647. doi:10.1074/jbc.272.41.25641. PubMed: 9325286. 9325286 10.1074/jbc.272.41.25641

[35] Ludtke JJ, Sebestyén MG, Wolff JA (2002) The effect of cell division on the cellular dynamics of microinjected DNA and dextran. Mol Ther 5: 579-588. doi:10.1006/mthe.2002.0581. PubMed: 11991749. 11991749 10.1006/mthe.2002.0581

[36] Dean DA, Byrd JN, Dean BS (1999) Nuclear targeting of plasmid DNA in human corneal cells. Curr Eye Res 19: 66-75. doi:10.1076/ceyr.19.1.66.5344. PubMed: 10415459. 10415459 10.1076/ceyr.19.1.66.5344

[37] Escriou V, Ciolina C, Helbling-Leclerc A, Wils P, Scherman D (1998) Cationic lipid-mediated gene transfer: analysis of cellular uptake and nuclear import of plasmid DNA. Cell Biol Toxicol 14: 95-104. doi:10.1023/A:1007425803756. PubMed: 9553720. 9553720 10.1023/a:1007425803756

[38] Lechardeur D, Sohn KJ, Haardt M, Joshi PB, Monck M et al. (1999) Metabolic instability of plasmid DNA in the cytosol: a potential barrier to gene transfer. Gene Ther 6: 482-497. doi:10.1038/sj.gt.3300867. PubMed: 10476208. 10476208 10.1038/sj.gt.3300867

[39] Zelphati O, Liang X, Hobart P, Felgner PL (1999) Gene chemistry: functionally and conformationally intact fluorescent plasmid DNA. Hum Gene Ther 10: 15-24. doi:10.1089/10430349950019156. PubMed: 10022527. 10022527 10.1089/10430349950019156

[40] Pollard H, Toumaniantz G, Amos JL, Avet-Loiseau H, Guihard G et al. (2001) Ca2+-sensitive cytosolic nucleases prevent efficient delivery to the nucleus of injected plasmids. J Gene Med 3: 153-164. doi:10.1002/jgm.160. PubMed: 11318114. 11318114 10.1002/jgm.160

[41] Banks GA, Roselli RJ, Chen R, Giorgio TD (2003) A model for the analysis of nonviral gene therapy. Gene Ther 10: 1766-1775. doi:10.1038/sj.gt.3302076. PubMed: 12939643. 12939643 10.1038/sj.gt.3302076

