# Supplementary figures and images for: In Vitro Efficient Transfection by CM18-Tat11 Hybrid Peptide: A New Tool for Gene-Delivery Applications
Source: PLoS One. 2013 Jul 29;8(7):e70108. doi: 10.1371/journal.pone.0070108 (PMC3726494; doi:10.1371/journal.pone.0070108)

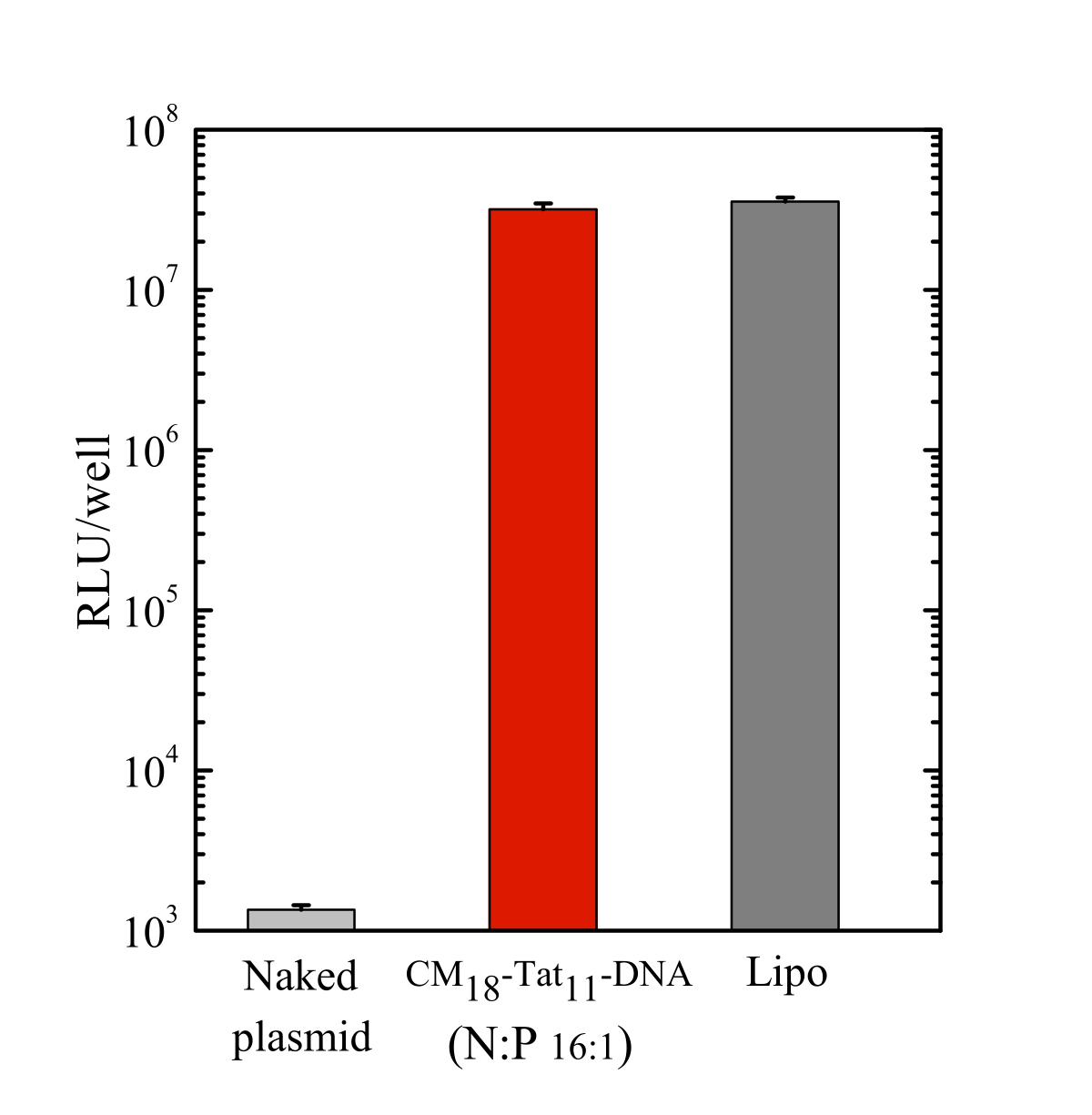

Supplement: Figure S1 — Transgene expression is detected 24 hours after transfection by measuring luciferase activity from an aliquot of the CHO cells external medium. Light grey column is the mean value obtained with naked DNA, dark grey column is for lipofectamine, while red column is for CM18-Tat11/DNA complex at N:P ratio 16:1. The reported RLU/well values represent the mean of three independent measurements, each performed in triplicate. (TIFF) [file pone.0070108.s001.tiff]

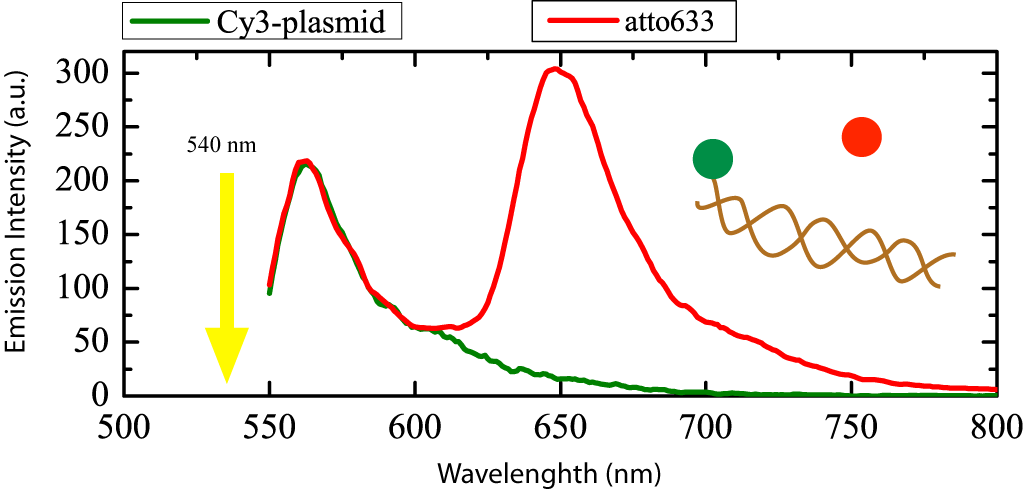

Supplement: Figure S2 — Fluorescence spectra are recorded at 37 °C with a spectrofluorometer by exciting at 540 nm and collecting the fluorescence between 550 and 800 nm. First, a fluorescence emission measurement is performed on a PBS solution of Cy3-labeled DNA plasmid alone (solid green line). Then, isolated atto633 (solid red line) at N:P ratio 16:1 is added, and the emission spectrum recorded again. No donor quenching is detected. (TIF) [file pone.0070108.s002.tif]

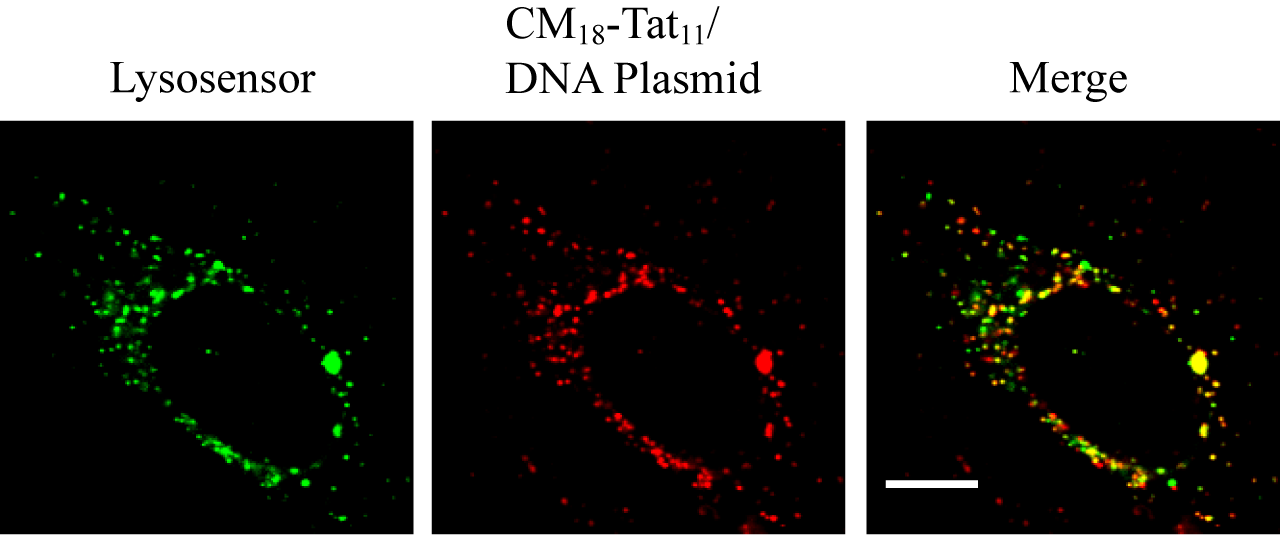

Supplement: Figure S3 — Colocalization of Lysosensor signal (lysosome marker, green) with atto633-CM18-Tat11/DNA 16:1 complex signal (red) after 12 h of treatment. The overlay (yellow) reveals that the complex is completely delivered to the lysosomal compartment. (TIF) [file pone.0070108.s003.tif]
